# Abdominal Pedicled Flap: Alternative for Hand Reconstruction When Microsurgery Is Not an Option

**DOI:** 10.7759/cureus.84969

**Published:** 2025-05-28

**Authors:** Tirzo Augusto Aguilar Matias, Bryan A Morales Eslava, María del Carmen Garduño Pérez

**Affiliations:** 1 Plastic and Reconstructive Surgery, Universidad Nacional Autónoma de México, Mexico City, MEX; 2 General Surgery, Universidad Juárez Autónoma de Tabasco, Villahermosa, MEX

**Keywords:** abdominal flap, distally based hand flap, hand reconstruction, microsurgery, plastics reconstruction, random pattern flaps, soft tissue coverage, soft tissue defect, staged reconstruction

## Abstract

Soft tissue defects of the dorsal hand, especially overlying tendons and other important structures, can prove to be a reconstructive challenge; though microsurgical free flaps are considered the gold standard, the lack of resources often necessitates alternative approaches. A 74-year-old male patient presented with dorsal hand loss caused by skin infection and abscess formation after prolonged use of an antebrachiopalmar splint, resulting in an 18 cm × 12 cm defect with tendon exposure and fibrin deposition. A random-pattern pedicled abdominal flap harvested from the infraumbilical right lower abdominal quadrant was transposed to the defect and secured with simple nylon 3-0 sutures, while the donor site was primarily closed. Complete functional recovery without complications was achieved after pedicle division at three weeks. Random-pattern pedicled abdominal flaps should thus be regarded as a valuable staple in upper extremity reconstruction, particularly when microsurgery is not feasible.

## Introduction

Immediate and stable reconstruction of soft tissue defects of the hand with exposure of tendons, nerves, or bone is required to prevent functional impairment. Local, regional, distant, and microsurgical free flap reconstructive techniques have been described. While microsurgical techniques are considered the gold standard for reconstruction due to their individualized approach, their application is often limited by institutional resources, patient comorbidities, or vascular contraindications. In these cases, conventional methods such as pedicled abdominal flaps still present an option. We report a case of dorsal hand reconstruction with an abdominal random-pattern pedicled flap where the microsurgical option was not available.

An abdominal pedicled flap is a regional flap harvested from the lower abdomen while maintaining its vascular pedicle, allowing tissue transfer to the upper extremity without the need for microsurgical anastomosis. It is most often based on perforators from the superficial inferior epigastric artery or other abdominal vessels, such as the superficial circumflex iliac and superficial external pudendal arteries [1]. These flaps are typically indicated in cases involving large soft tissue defects of the hand or forearm, particularly when microsurgical facilities are unavailable or when patient-specific factors preclude free flap use [2,3].

Reconstructive alternatives include skin grafts, local or regional fasciocutaneous or musculocutaneous flaps, and microsurgical free tissue transfer. Among these, microsurgery offers the most precise, adaptable coverage with superior aesthetic and functional outcomes, especially for small to moderate-sized defects [2]. However, microsurgical reconstruction requires a highly trained team, suitable recipient vessels, and prolonged operative and ischemia times, limiting its widespread applicability [4].

In contrast, abdominal pedicled flaps offer a simpler, time-tested technique with reliable vascularity and relatively easier execution, especially in low-resource settings. However, disadvantages include the need for patient immobilization during the attachment phase, risk of joint stiffness, potential bulkiness of the flap, and staged surgical interventions requiring subsequent flap division [5,6].

Several studies have demonstrated comparable functional results between abdominal pedicled flaps and microsurgical reconstructions, particularly in centers lacking microsurgical infrastructure. For instance, Jabaiti et al. reported an 85.3% flap survival rate with random abdominal flaps, while Jaramillo et al. achieved 100% survival with vertical-pattern abdominal flaps in hand reconstruction [4,7]. These findings underscore the continued relevance of pedicled abdominal flaps in the reconstructive armamentarium, particularly when tailored to individual patient and institutional contexts.

When addressing soft tissue defects of the hand, flap selection must balance defect size, donor site morbidity, patient comorbidities, and surgical expertise. The abdominal pedicled flap, often used when microsurgical resources are limited, offers a straightforward, low-risk option, particularly in severe injuries or infection-prone environments. However, it demands prolonged immobilization and poses aesthetic and functional limitations due to joint stiffness and lack of sensibility. In contrast, the pedicled radial forearm flap is a versatile local option that provides thin, pliable skin with good vascular reliability. It allows single-stage coverage without microvascular anastomosis, although at the cost of potential donor site morbidity and poor aesthetic outcome in some cases. On the other hand, the free anterolateral thigh (ALT) flap has become a microsurgical gold standard due to its long pedicle, large skin territory, and low donor morbidity when a septocutaneous perforator is present. It is best suited for large or complex defects, provided a trained microsurgical team and adequate recipient vessels are available.

This case report adds to the growing body of evidence that supports the use of abdominal pedicled flaps as a dependable option in upper limb reconstruction, offering functional recovery and aesthetic restoration in the absence of microsurgical capability.

## Case presentation

A 74-year-old male patient with no significant past medical history presented with ulcerations to the dorsal surface of the right arm and hand secondary to an underlying soft tissue infection and abscess formation due to prolonged use of an antebrachiopalmar splint. The patient was initially managed by the orthopedic trauma service and later received integrated care involving orthopedic surgery, infectious diseases, and reconstructive surgery. Tissue cultures obtained confirmed Staphylococcus aureus sensitive to vancomycin, for which a 14-day course of intravenous vancomycin was initiated and monitored by the infectious diseases team.

Initial surgical findings

The orthopedic trauma team first performed surgical intervention, revealing a dorsal hand abscess with extension of surrounding soft tissue involvement to the wrist. A tunneling defect was observed in the carpal area, extending to extensor zones 7 and 8. Tissues involved were devitalized, with only poor vascularization and a penchant for friability, requiring extensive debridement.

Reconstructive surgery findings

**Figure 1:**
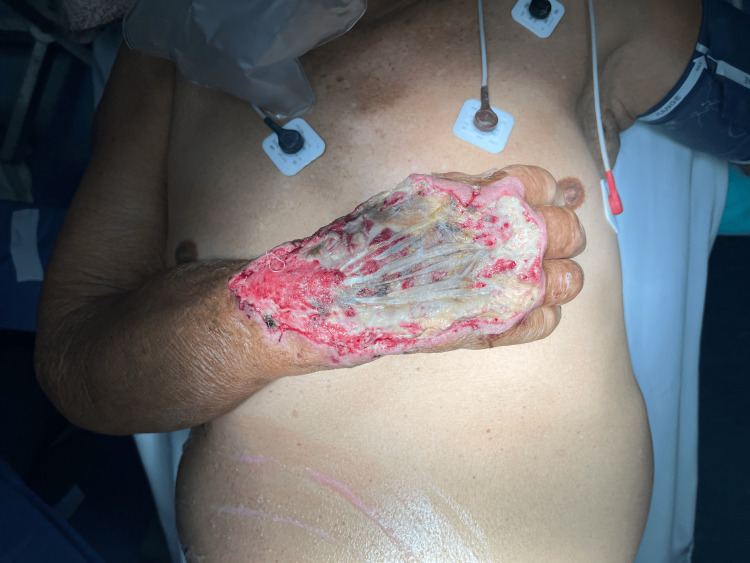
An 18 cm × 12 cm soft tissue defect on the dorsal aspect of the right hand, with exposed extensor tendons and fibrin deposition within the wound bed

After primary debridement, the reconstructive surgery team was consulted to assess the patient, and a total soft tissue defect of 18 cm × 12 cm on the dorsum of the right hand with exposed extensor tendons and fibrin deposit within the wound bed was noted. Due to the size of the defect and the requirement for lasting coverage, reconstruction via a random-pattern pedicled abdomen flap was planned (Figure 1).

Surgical technique

**Figure 2:**
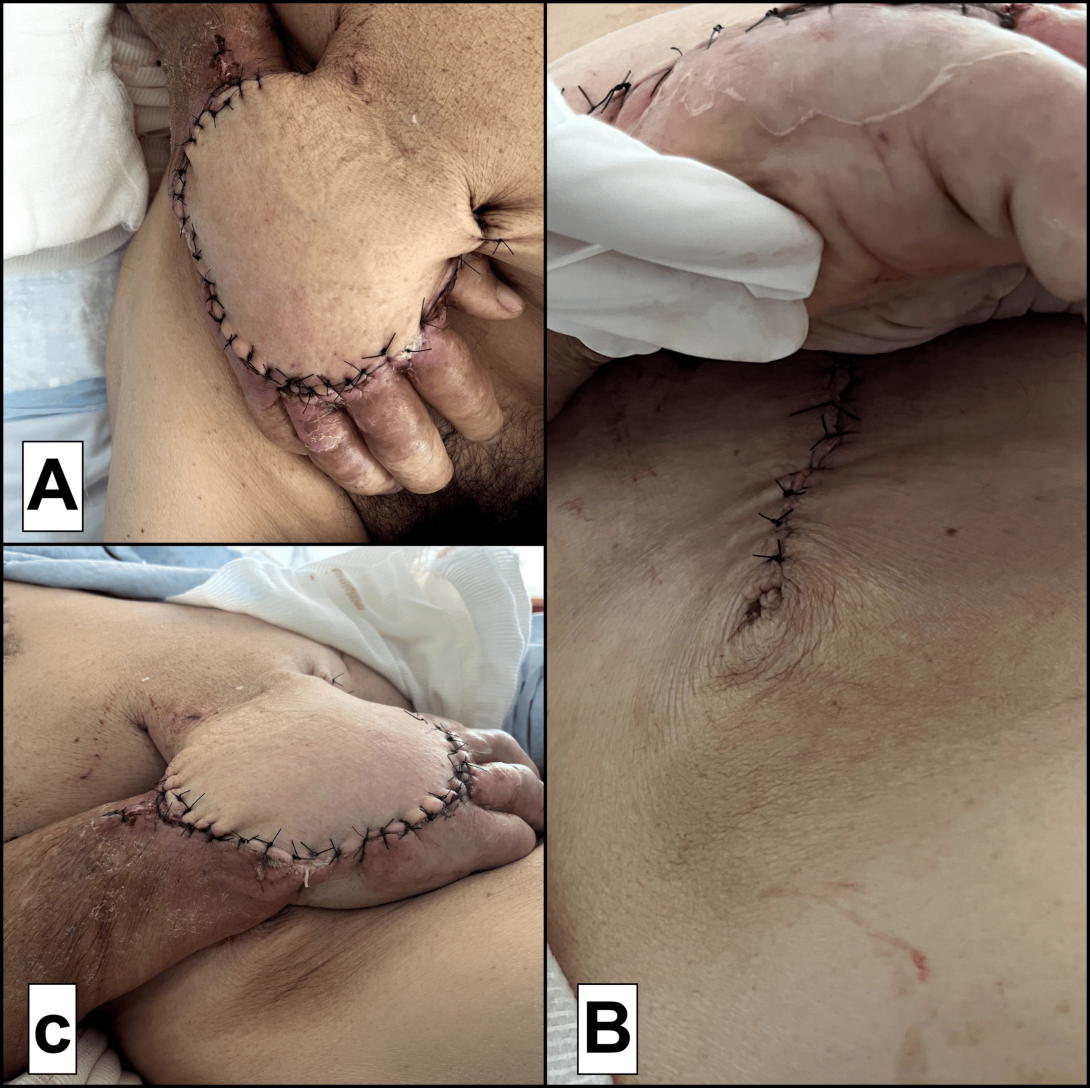
Immediate postoperative results (A) Immediate postoperative view showing the hand inset into the infraumbilical abdominal donor site after elevation of the random-pattern pedicled flap, with secure flap fixation and good flap perfusion; (B) Close-up of the abdominal incision showing primary closure of the donor site, with well-approximated skin edges and no signs of tension or dehiscence; (C) Lateral view of the hand attached to the abdominal wall, demonstrating the bulk and full inset of the flap with proper alignment and tension-free adaptation.

The wound was debrided under general anesthesia, including necrotic tissue removal and optimization of the recipient bed. A random-pattern abdominal flap was designed from the infraumbilical region of the right lower abdominal quadrant with sufficient vascular supply. The flap was elevated and transposed without tension to the dorsal hand defect. The flap was secured to the recipient site with simple nylon 3-0 sutures. The donor was closed mostly in deep layers using inverted Vicryl 3-0 sutures and skin with simple nylon 3-0 sutures (Figure 2).

Postoperative course

**Figure 3:**
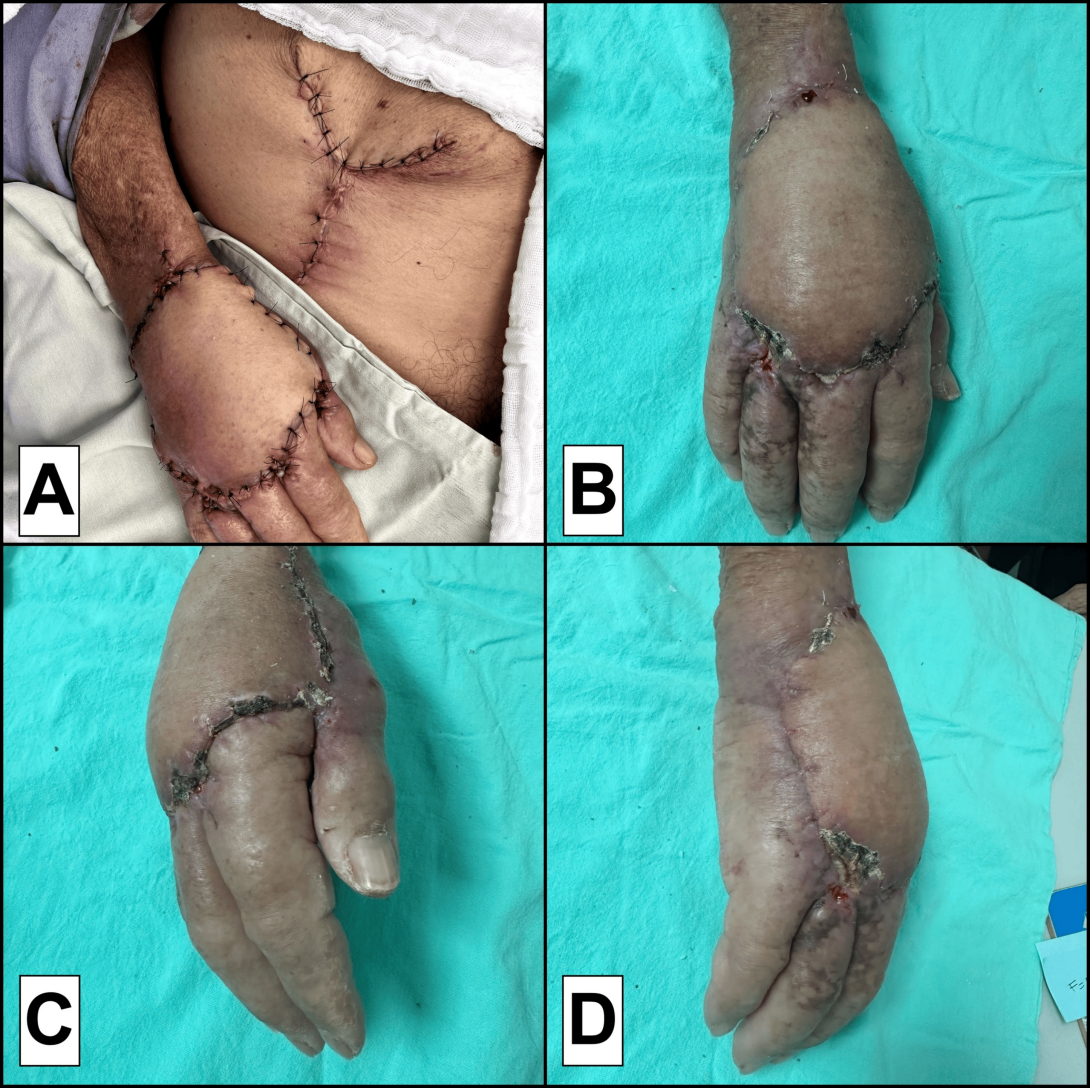
Flap successfully integrated (A) Immediate postoperative view after pedicle division and final insetting of the abdominal flap, showing adequate perfusion and flap viability; (B–D) Progressive views of the fully integrated abdominal flap at different postoperative stages, demonstrating good healing, contour adaptation, and absence of major complications.

Postoperatively, the patient was positioned to minimize tension on the flap during the initial postoperative period. Daily monitoring confirmed normal perfusion and no evidence of ischemia or necrosis. Three weeks later, the pedicle was divided, and the final insetting was performed. The flap was successfully incorporated by the follow-up period, with minimal complications and acceptable functional and aesthetic results (Figure 3). The patient had fully restored mobility and grip function and was referred for rehabilitation and optimization of hand mobility.

## Discussion

Soft tissue defects in the hand and upper extremities that require turnover coverage remain a complex challenge because the underlying tendons, nerves, and bones are often exposed, and durable, stable tissue coverage is needed [1]. Reconstruction techniques may include local and regional flaps, as well as complex microsurgical free tissue transfers.

Although the gold standard in complex upper extremity reconstruction, free flaps are often limited in practice due to resource availability, patient comorbidities, and/or suboptimal recipient vessels [2,3]. The abdominal pedicled flaps remain valid reconstructive alternatives in these situations, as outlined by several studies, especially in extensive wounds and severe burns [8,9].

Flaps are especially useful due to their inherent versatility, which is primarily supported by reliable vascular supplies from the deep inferior epigastric artery, superficial circumflex iliac artery, and superficial external pudendal artery [9], which are referred to as abdominal pedicled flaps. This anatomical pliability enables flap designs tailored based on the hand defect's nature and extent and is especially useful for complex hand injuries, digit amputations, or extensive avulsion injuries that necessitate vigorous and abundant coverage [5,8,9].

To further illustrate the technical aspects and comparative indications of abdominal pedicled flaps and alternative flap options, we provide detailed visual references and highlight the practical advantages and limitations inherent to each technique.

**Figure 4:**
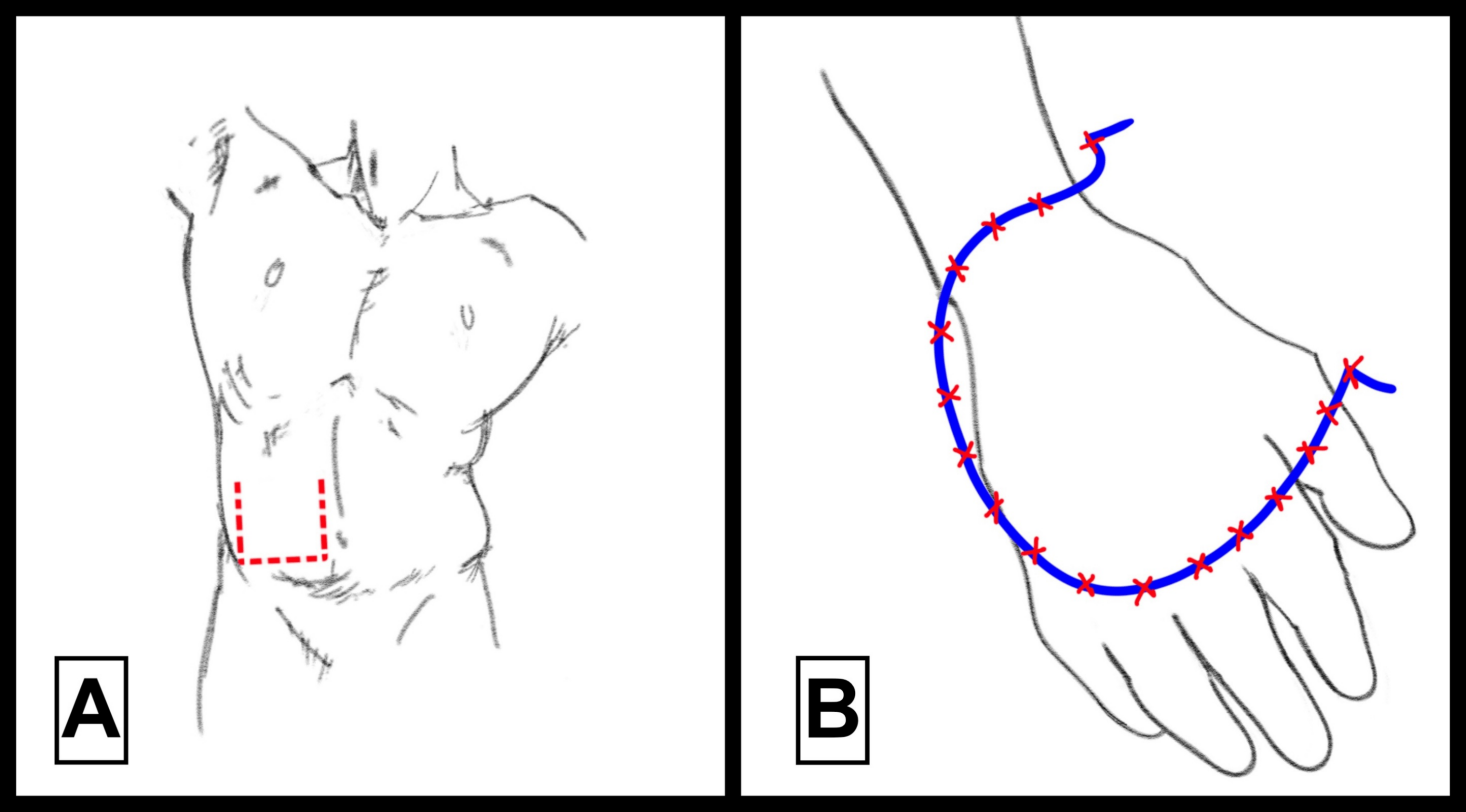
Surgical images illustrating the pedicled abdominal pocket flap technique All surgical techniques illustrated are adapted from images from [10].

The pocket flap represents a reliable option for extensive hand defects when microsurgical reconstruction is unavailable or unsuitable. The procedure begins with the delineation and elevation of an abdominal flap based on local vascular patterns, as illustrated in Figure 4A, ensuring the viability of the transferred tissue [10]. Subsequently, the affected hand is strategically inserted into the subcutaneous abdominal pocket prepared beforehand with proper tension and secure fixation using simple sutures (Figure 4B). The abdominal pocket’s position is meticulously planned to maximize patient comfort and reduce vascular complications, maintaining optimal blood supply to the flap [10]. After approximately three weeks, once flap viability is confirmed, the final separation and definitive coverage of the defect are performed, thus achieving functional and aesthetic restoration of the affected region [10].

**Figure 5:**
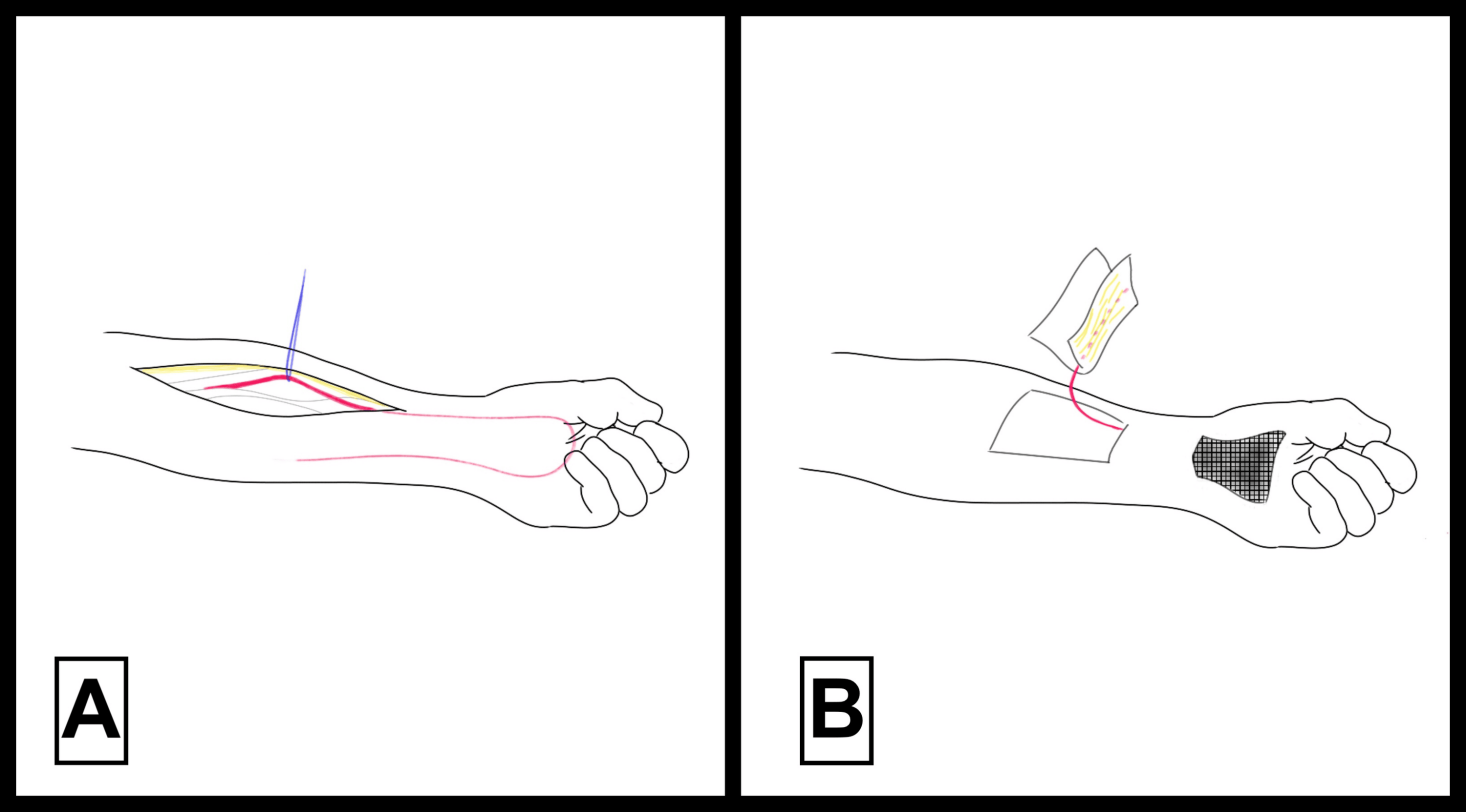
Surgical image illustrating the pedicled radial forearm flap technique All surgical techniques illustrated are adapted from [10].

The pedicled radial forearm flap, shown in Figure 5A, involves the elevation of a flap with the radial artery and its accompanying veins (Figure 5B) [10]. It offers strong vascularity and pliability, along with favorable pedicle length and tissue characteristics. However, donor-site morbidity and the necessity for secondary skin grafting to cover the donor site are significant limitations [10].

**Figure 6:**
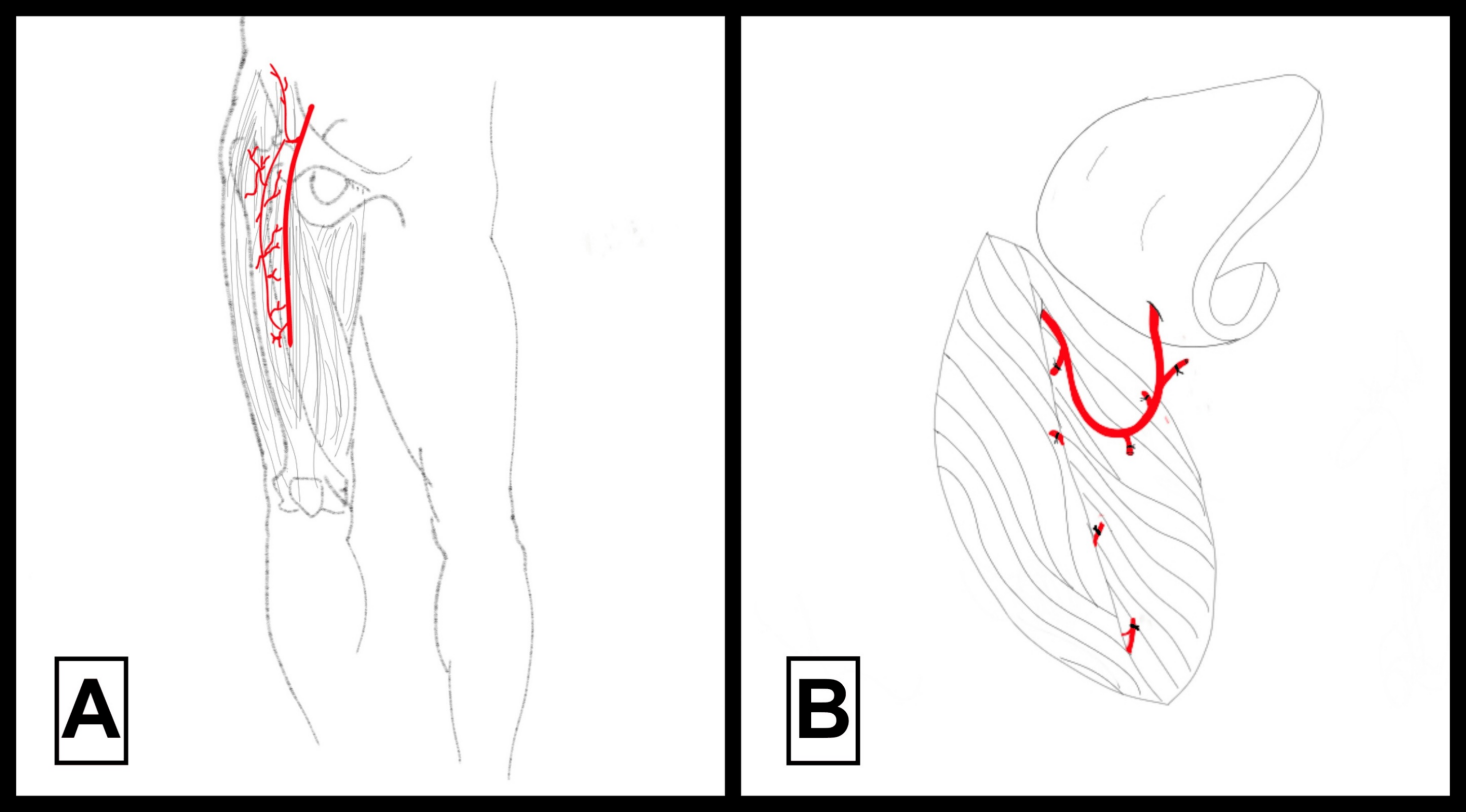
Surgical images illustrating the free anterolateral thigh (ALT) flap technique All surgical techniques illustrated are adapted from [10].

The free ALT flap, illustrated in Figure 6, provides substantial surface area and reliable perfusion for reconstruction purposes, owing to its substantial size and reliable blood supply [10]. The surgical technique entails meticulous dissection and isolation of perforating vessels from the thigh for microsurgical transfer to the recipient area (Figures 6A, 6B). Benefits include the flap’s substantial surface area coverage and excellent functional and aesthetic outcomes. Nonetheless, limitations involve technical complexity, prolonged surgical duration, and inherent risks associated with microsurgical vessel anastomosis, requiring significant surgical expertise and technical proficiency [10].

The survival rates and functional outcomes of random-pattern abdominal pedicled flaps have remained admirably high. Jabaiti et al., in a series of 34 patients, reported an 85.3% flap survival rate, as 11.8% experienced partial necrosis and only 2.9% complete flap loss [7]. Similarly, Jaramillo et al. showed a 100% survival rate in 10 patients using vertical-pattern abdominal flaps with a low complication rate [4]. These results are consistent with our present case, where the flap integrated successfully and allowed for full restoration of hand function without significant complications.

Three clinical situations in particular highlight the continuing relevance of abdominal pedicled flaps: when defects are too extensive for local flaps; when microsurgical settings or expertise are unavailable; and when suitable recipient vessels are compromised by trauma or previous surgery [1, 3, 9].

In our patient, the decision of a random-pattern abdominal pedicled flap was mainly related to the lack of microsurgery service, indicating practical limitations that affect the universal utility of microsurgical techniques. Thus, proficient use of conventional reconstructive procedures is still essential, especially in low-resource areas [4, 6].

Nevertheless, careful selection of patients and meticulous surgical planning are still critical. The structural bulkiness of abdominal flaps may also necessitate secondary debulking for both functional and aesthetic improvement [4]. In our experience, an intraoperative thinning effectively mitigated the need for future surgeries.

Furthermore, joint stiffness, a known complication of the immobilization of the limb tethered to the abdominal donor site (reported by Acharya et al. in as many as 39% of patients in their study), remains a conceptually unexplored side effect of this form of reconstruction. Importantly, prompt prophylactic joint positioning ("James position") and early physiotherapy alleviated this complication and resulted in good functional recovery [9, 11].

The staged surgical approach inherent in pedicled flaps, which requires the division of that flap after a period of delay, can also carry risks of marginal necrosis or delayed wound healing [1, 8, 9]. This approach demands precise surgical technique and strict postoperative observation, which were vital in attaining complete viability in our patient.

Modern innovations and technological refinements in abdominal flap creation have attempted to overcome these classic challenges. Pivoted abdominal flaps using perforator-based designs with deep inferior epigastric artery perforator (DIEP) flaps yield superior flexibility, vascular reliability, and lower necrosis rates, even with large defects extending to the distal forearm territory [12, 13].

Moreover, rehabilitation protocols during the abdominal attachment phase that started earlier have also been emphasized, leading to better functional outcomes and less postoperative stiffness [8,12]. Long-term functional outcomes with pedicled abdominal flaps are similarly encouraging. Recent comparisons have shown that properly performed pedicled abdominal flaps achieve similar functional results to those of microsurgical free flaps. Acharya et al. reported that at one-year follow-up, there existed minimal functional disability (low Disabilities of the Arm, Shoulder, and Hand (DASH) scores), thus supporting that pedicled abdominal flaps, by utilizing technically safe surgical protocols, can perform comparably to their microsurgical counterparts regarding functional recovery [5]. Sabapathy et al. [1] described similar results, with emphasis that the safety and effectiveness of pedicled abdominal flaps must not be despised because we all know that there are some specific contexts, such as high-voltage electrical burns or pediatric reconstructions, where abdominal flaps can only be decided.

In this respect, it should be borne in mind that, despite the known advances in volumetric techniques of microsurgical flaps, the use of abdominal pedicled flaps retains its relevance and complementary role in reconstructive surgery of complex hand defects. When patient selection is judicious and surgical techniques are refined, surgical outcomes with these flaps are reliable and functionally and cosmetically satisfactory, especially in the resource-limited setting.

## Conclusions

This case demonstrates the success of random-pattern pedicled abdominal flaps for reconstruction of large dorsal hand defects, especially in centers where microsurgical techniques are not available or contraindicated. The good outcome demonstrated in our patient, with total mobility and functional recovery, is consistent with what is described in the literature. Future studies should continue to focus on patient selection criteria and surgical techniques to further improve long-term outcomes.
